# Differential *In Vitro* Cultivation of *Francisella tularensis* Influences Live Vaccine Protective Efficacy by Altering the Immune Response

**DOI:** 10.3389/fimmu.2018.01594

**Published:** 2018-07-10

**Authors:** Raju Sunagar, Sudeep Kumar, Sarah J. Rosa, Karsten R. O. Hazlett, Edmund J. Gosselin

**Affiliations:** Department of Immunology and Microbial Disease, Albany Medical College, Albany, NY, United States

**Keywords:** *Francisella tularensis*, live vaccine, brain hear infusion medium, biodefense, mucosal vaccines

## Abstract

*Francisella tularensis* (*Ft*) is a biothreat agent for which there is no FDA-approved human vaccine. Currently, there are substantial efforts underway to develop both vaccines and improved tools to assess these vaccines. *Ft* expresses distinct sets of antigens (Ags) *in vivo* as compared to those expressed *in vitro*. Importantly, *Ft* grown in brain-heart infusion medium (BHIM) more closely mimics the antigenic profile of macrophage-grown *Ft* when compared to Mueller-Hinton medium (MHM)-grown *Ft*. Thus, we predicted that when used as a live vaccine BHIM-grown *Ft* (BHIM-*Ft*) would provide better protection, as compared to MHM-*Ft*. We first determined if there was a difference in growth kinetics between BHIM and MHM-*Ft*. We found that BHIM-*Ft* exhibited an initial growth advantage *ex vivo* that manifests as slightly hastened intracellular replication as compared to MHM-*Ft*. We also observed that BHIM-*Ft* exhibited an initial growth advantage *in vivo* represented by rapid bacterial expansion and systemic dissemination associated with a slightly shorter mean survival time of naive animals. Next, using two distinct strains of *Ft* LVS (WT and *sodB*), we observed that mice vaccinated with live BHIM-*Ft* LVS exhibited significantly better protection against *Ft* SchuS4 respiratory challenge compared to MHM-*Ft*-immunized mice. This enhanced protection correlated with lower bacterial burden, reduced tissue inflammation, and reduced pro-inflammatory cytokine production late in infection. Splenocytes from BHIM-*Ft sodB*-immunized mice contained more CD4^+^, effector, memory T-cells, and were more effective at limiting intracellular replication of *Ft* LVS *in vitro*. Concurrent with enhanced killing of *Ft* LVS, BHIM-*Ft sodB*-immune splenocytes produced significantly higher levels of IFN-γ and IL-17A cytokines than their MHM-*Ft sodB*-immunized counterparts indicating development of a more effective T cell memory response when immunizing mice with BHIM-*Ft*.

## Introduction

In addition to the antigen (Ag)/immunogen utilized, a number of other key factors influence vaccine efficacy, which include: bacterial strain (1, 2), growth conditions of the attenuated or killed vaccine, challenge strain (3–5), as well as the genetic background (6, 7), and gender of the host (8–10). Regarding bacterial growth conditions, immunogens used as attenuated or killed vaccines must first be grown *in vitro*. Studies have clearly demonstrated that the protective ability of such vaccines are dictated in part by the medium in which the vaccine candidate is generated with the choice of medium potentially altering the antigenic composition and thus efficacy of whole cell-based attenuated and killed vaccines (3, 5, 11). Specifically, the *in vitro* culture medium utilized to generate whole cell-based vaccines can impact the expression of antigenic determinants, pathogen-associated molecular patterns, and virulence factors expressed by the infectious agent. For example, many microbes have been reported to differentially express immunogenic molecules dependent on growth medium (12–16). Specifically, in a study of whole cell-based vaccines involving BCG, differential cultivation of BCG in Sauton versus Middlebrook medium significantly altered its protective efficacy and the enhanced protection generated by Middlebrook-derived BCG was associated with higher numbers of Mycobacteria-specific T helper type 17 (TH17) cells and higher Ab levels (17).

In regard to *Francisella tularensis* (*Ft*) specifically, the most commonly used media for cultivation are Mueller-Hinton medium (MHM), Chamberlain’s Defined medium (CDM), or brain-heart infusion medium (BHIM) (3, 11, 18). *Ft* grown *in vitro* in MHM (MHM-*Ft*) expresses a distinct set of genes as compared to those obtained from tissues or MØs following *Ft* infection (3, 4, 11). Whereas, *Ft* grown in BHIM *in vitro* (BHIM-*Ft*) produces a protein expression and pro-inflammatory cytokine pattern *in vivo* more closely resembling that of *Ft* obtained from macrophages *in vivo* (3, 4, 11). Further, differences between MHM-*Ft* versus BHIM-*Ft* can be attributed to differential protein and surface carbohydrate expression, as well as differences in structural integrity (11). The altered protein and carbohydrate expression and structural integrity of MHM-*Ft* versus BHIM-*Ft* can then lead to differences in their ability to interact with complement and *Ft* LPS-specific Abs, with MHM-*Ft* being more reactive (11). In addition, using *Ft* grown in BHIM or MHM as models of host-adapted and non-host-adapted bacteria respectively, we showed that *Ft* LVS and *Ft* SchuS4 grown in BHIM accumulate more capsular material that hampers the various immune effectors from interacting with it, leading to a shortened median survival time of naive mice challenged with *Ft* SchuS4 (3). Our recent study has also demonstrated that BHIM-grown *Ft* SchuS4 is more virulent when administered to *Ft* LVS-immunized mice versus MHM-grown *Ft* SchuS4 (4). Similarly, evaluation of the impact of growth medium on aerosolization and infectivity of *Ft* LVS *via* an aerosol challenge model demonstrated that growth of *Ft* SchuS4 in BHIM was associated with increased bacterial survival during aerosolization and a decreased LD_99_ (19). Collectively, the above findings indicate that *Ft* infection of naive or vaccinated animals can be impacted considerably by the immediate growth history of the pathogen. Similarly, the influence of growth media on tularemia vaccination has also been demonstrated. Specifically, in the case of inactivated vaccine, we reported that the immune-stimulatory nature of MHM-grown inactivated *Ft* (i*Ft*) generates better protection against *Ft* LVS challenge (5). In contrast, our preliminary studies using live *Ft* vaccination indicate BHIM-*Ft* LVS*-*immunized mice are better protected against *Ft* SchuS4 mucosal challenge than that of live *Ft* LVS grown in MHM (8). This contrasting observation may be due, in part, to the distinct immunological requirements for protection against *Ft* LVS and *Ft* SchuS4 or live versus inactivated vaccine. The latter is supported by the observations that BHIM-grown *Ft* organisms express higher levels of immunodominant Ags, virulence factors, and surface-carbohydrate synthases *via FevR* regulon expression and thus are more virulent for naive, as well as vaccinated mice, as compared to MHM-grown *Ft* (4).

In this study, we sought to expand our analysis of the influence of growth medium on the efficacy of live *Ft* vaccine. We hypothesized that the efficacy of *Ft* live vaccine would be impacted by the growth medium in which *Ft* is propagated. We demonstrate that *Ft* grown in BHIM is a more protective vaccine than MHM-grown *Ft*, following *Ft* SchuS4 challenge. Furthermore, changes in bacterial burden, tissue damage, inflammation, Ab response, and recall response, correlates with improved vaccine efficacy of live BHIM-*Ft* versus that of MHM-*Ft*.

## Materials and Methods

### Mice

Specific pathogen-free, C57BL/6 male and female mice 6–8 weeks of age were purchased from Taconic Farms (Hudson, NY, USA). Mice were housed in sterile microisolator cages in the animal biosafety level 2 and ABSL-3 facilities at the Albany Medical Center. All animal studies were reviewed and approved by the Institutional Animal Care and Use Committee at Albany Medical College according to NIH standards.

### Bacteria

*Francisella tularensis* holartica LVS (*Ft* LVS) and *Ft* LVS superoxide dismutase *sod* B mutant were cultured in MHM or BHIM on agar plates (Becton Dickinson, Sparks, MD, USA) at 37°C for 48 h and subsequently used for vaccination. The challenge pathogen *Ft* SchuS4 was cultured aerobically in MHM or BHIM broth (Becton Dickinson, Sparks, Maryland) and the active mid-log phase bacteria were harvested and used for mouse infection (8, 9). Inactivated *Ft* (i*Ft*) was generated, as previously described (5). For *in vivo* imaging studies, *Ft* LVS was transformed with the luminescence reporter plasmid (pXB173-lux), as described previously by Bina et al. (20). Kanamycin (km) was used at 50 µg/ml to maintain selection for *Ft* bearing the lux-reporter plasmid.

### Whole-Animal Luminescent Imaging

The photon emissions from mice that had been infected with *Ft* LVS-lux were measured using an *in vivo* imaging system (IVIS) Lumina whole live-animal imaging system (Caliper Life Sciences, Hopkinton, MA, USA). Mice were anesthetized with isoflurane using a precision vaporizer and oxygen before and during imaging. Images presented in this study were acquired using a field view of C or D, a maximum auto-exposure time of 5 min, a binning factor of 4, and an f/stop of 1. Bioluminescence within specific regions of individual animals was quantified using the region-of-interest (ROI) tool in Living Image software, version 4.5 (PerkinElmer). The relative intensities of transmitted light from bioluminescence and scales were determined automatically and reported as photons/s/cm^2^/sr.

### Immunization and Challenge Studies

Mice were anesthetized with an intraperitoneal (i.p.) injection of 100 µl of 20 mg/ml ketamine (Vedco, St. Joseph, MO, USA) and 0.4 mg/ml xylazine (Lioyd, Shenandoah, Iowa). Mice were subsequently immunized, as previously described (9). Specifically, 1 × 10^3^ CFU of BHIM or MHM-*Ft* LVS or -*Ft sodB* were administered intradermally (i.d.) in 50 µl of PBS followed by an i.n. boost with 1 × 10^3^ CFU in 20 µl of PBS on day 21 post-primary immunization. Mice were then challenged i.n. with frozen stocks of MHM or BHIM-*Ft* SchuS4 (20–200 CFU) on day 42 post-immunization. In all cases, challenged mice were subsequently monitored for survival for a minimum of 30 days using death as an endpoint.

### Quantification of Bacterial Burden

Mice were sacrificed by i.p. injection of pentobarbital (Fort Dodge Laboratories), followed by cervical dislocation at various time intervals, as indicated in the individual figures. The bacterial burden in the lungs, liver, and spleen of infected mice was monitored, as previously described (21). Briefly, tissues from infected mice were collected aseptically in PBS containing a protease inhibitor mixture [Complete EDTA-free protease inhibitor cocktail tablets (Roche Diagnostics, IN)] and tissues were subjected to mechanical homogenization using a MiniBeadBeater-8 with 1-mm zirconia/silica beads (BioSpec Products, Bartlesville, OK, USA). Tissue homogenates were diluted 10-fold in sterile PBS and 10 µl of each dilution was spotted onto chocolate agar plates (Becton Dickinson, Sparks, MD, USA) in duplicate and incubated at 37°C for 2–3 days. The number of colonies on the plates were then counted and expressed as log_10_ CFU per ml for each respective tissue.

### Assessment of Humoral Immune Responses

One week after the second immunization, blood was collected from the sub-mandibular vein of vaccinated and unvaccinated mice. Anti-*Ft* Ab production in response to vaccination was measured by enzyme linked immunosorbent assay, as previously described (9).

### *In Vivo* Cytokine Production

Tissue homogenates were obtained as indicated above when measuring bacterial burdens. Supernatants were then collected and stored at −20°C for cytokine analysis. Luminex assay was performed to determine *in vivo* cytokine levels of interferon-γ (IFN-γ), interleukin-6 (IL-6), interleukin-10 (IL-10), interleukin-17 (IL-17A), interleukin-12p40 (IL-12p40), tumor necrosis factor-α (TNF-α), and monocyte chemoattractant protein-1 (MCP-1) to assess inflammation.

### BMDM and Resident Peritoneal Macrophages (RPMs)

Bone marrow was flushed from femurs of naive C57BL6 mice with 2% heat-inactivated FBS (Hyclone, Logan, UT, USA) in PBS. A single-cell suspension was prepared, centrifuged, and erythrocytes were lysed with ammonium chloride. The cell pellet was resuspended in complete DMEM (Gibco, Grand Island, NY, USA) [cDMEM supplemented with d-glucose (4.5 mg/liter), l-glutamine (4 mM), and supplemented with 10% heat-inactivated FBS, 50 µM of β-mercaptoethanol, and recombinant murine macrophage colony stimulating factor (rM-CSF; Biolegend, San Diegao, CA, USA) 20 ng/ml]. The cells from each femur (~5 × 10^6^ cells) were resuspended in 10 ml of DMEM and plated in a 10 cm Petri dish and incubated at 37°C in 5% CO_2_. After 2 days, the cultures were replenished with fresh cDMEM-containing rM-CSF (10 ng/ml). On day 8 cells were detached from flask and seeded onto a 48-well plate at a density of 1 × 10^5^ cells per well. Following overnight incubation, the BMDMs were used in the co-culture assay. Peritoneal exudate cells from naïve mice were isolated without elicitation, these RPMs were cultured with RPMI 1640 supplemented with 10% FBS following overnight incubation at 37°C in 5% CO_2_. The non-adherent cells were then removed by aspiration of medium. Adherent cells, which comprise >90% macrophages, were used for infection studies.

### Splenocyte Preparation

Single cell suspensions from spleen were prepared from vaccinated mice at 1-week post-boost. A single-cell suspension was prepared, centrifuged, and erythrocytes were lysed with ammonium chloride. The splenocytes from three mice were then used either in the co-culture assay or in the *in vitro* recall response assay. For recall response, splenocytes were cultured in 24-well plate (Costar Corning, Corning, NY, USA) at 5 × 10^5^ cells (200 μl)/well.

### BMDMs-Splenocyte Co-Culture and *In Vitro* Cytokine Production

BMDMs prepared from naïve mice were plated in 48-well plate (1 × 10^5^/well). *Ft* LVS organisms were diluted in antibiotic-free cDMEM and added to BMDMs at a multiplicity of infection (MOI) of 50. Following 2 h incubation, unbound bacteria were removed and the remaining extracellular bacteria were eliminated by incubating BMDMs with 20 µg/ml gentamicin–cDMEM for 1 h. Medium or i*Ft* (10 i*Ft* per splenocyte) splenocytes from the unvaccinated or vaccinated mice were then added to the BMDMs at a ratio of 1:1.25 and incubated for 21 h at 37°C in 5% CO_2_. Culture supernatants were then collected at the indicated times and analyzed for IFN-γ, IL-17, IL-12p40, IL-10, IL-6, TNF-α, and MCP-1 using Luminex assay. Simultaneously, intracellular bacteria were enumerated by lysing cells at the indicated time points with 1% saponin (Sigma-Aldrich, St. Louis, MO, USA). Cell lysates were then serially diluted and plated on MHM agar plates and incubated at 37°C for 2–3 days before individual CFU were counted.

### Lactate Dehydrogenase (LDH) Release Assay

Serum concentrations of LDH were measured using a LDH activity assay kit (Sigma-Aldrich, St. Luis, Missouri), as previously described (9).

### CD4 and CD8 T Cell Frequencies

Splenocyte populations of vaccinated mice were obtained at 7 days post-boost and were assessed by flow cytometry, as previously described, using the following directly conjugated Abs, CD3 PerCP-Cy5.5 (clone17A2; Biolegend, San Diego, CA, USA), CD4 APC/Cy7 (clone GK1.5; eBioscience, San Diego, CA, USA), CD8 APC (clone 53–6.7; eBioscience, San Diego, CA, USA), CD44 PE-Cy7 (clone IM7; eBioscience, San Diego, CA, USA), and CD62L Alexa Fluor 488 (Clone MEL-14; Biolegend, San Diego, CA, USA). Subsequently, cells were washed and fixed on ice with 2% paraformaldehyde in PBS and the number of CD4 and CD8 T effector cells was then quantified using an LSRII flow cytometer.

### Intracellular Cytokine Staining

For intracellular cytokine staining, splenocytes from immunized mice were obtained at 7 days post-boost and stimulated overnight at 37°C in the absence or presence of BHIM or MHM-grown i*Ft* (1:25 ratio) in the presence of 5 µg/ml Brefeldin A (Biolegend, San Diego, CA, USA). Following incubation, the cells were stained with the cell surface marker Abs for CD3, CD4, and CD8, and then fixed with 2% paraformaldehyde. Cells were then resuspended in permeabilization buffer (BD Cytofix/Cytoperm kit; BD San Diego, CA, USA) for 30 min at 4°C and stained with anti-IFN-γ PE (clone XMG1.2, Biolegend, San Diego, CA, USA) Ab. Cells were washed thoroughly to remove residual unbound Ab. The number of CD4^+^ and CD8^+^ T cells positive for IFN-γ was then quantified using an LSRII flow cytometer.

### Statistical Analysis

Statistical data for bacterial clearance and cytokine production was generated using analysis of variances by two-way ANOVA or Mann–Whitney two-tailed test. In the case of survival, significance was determined using a log-rank (Montel–Cox) test. The data were analyzed using Graph-Pad prism (v6.0) software (Graph-Pad Software, San Diego, CA, USA).

## Results

### BHIM-*Ft* Displays Enhanced Replication, Dissemination, and Virulence *In Vivo*

**Figure 1:**
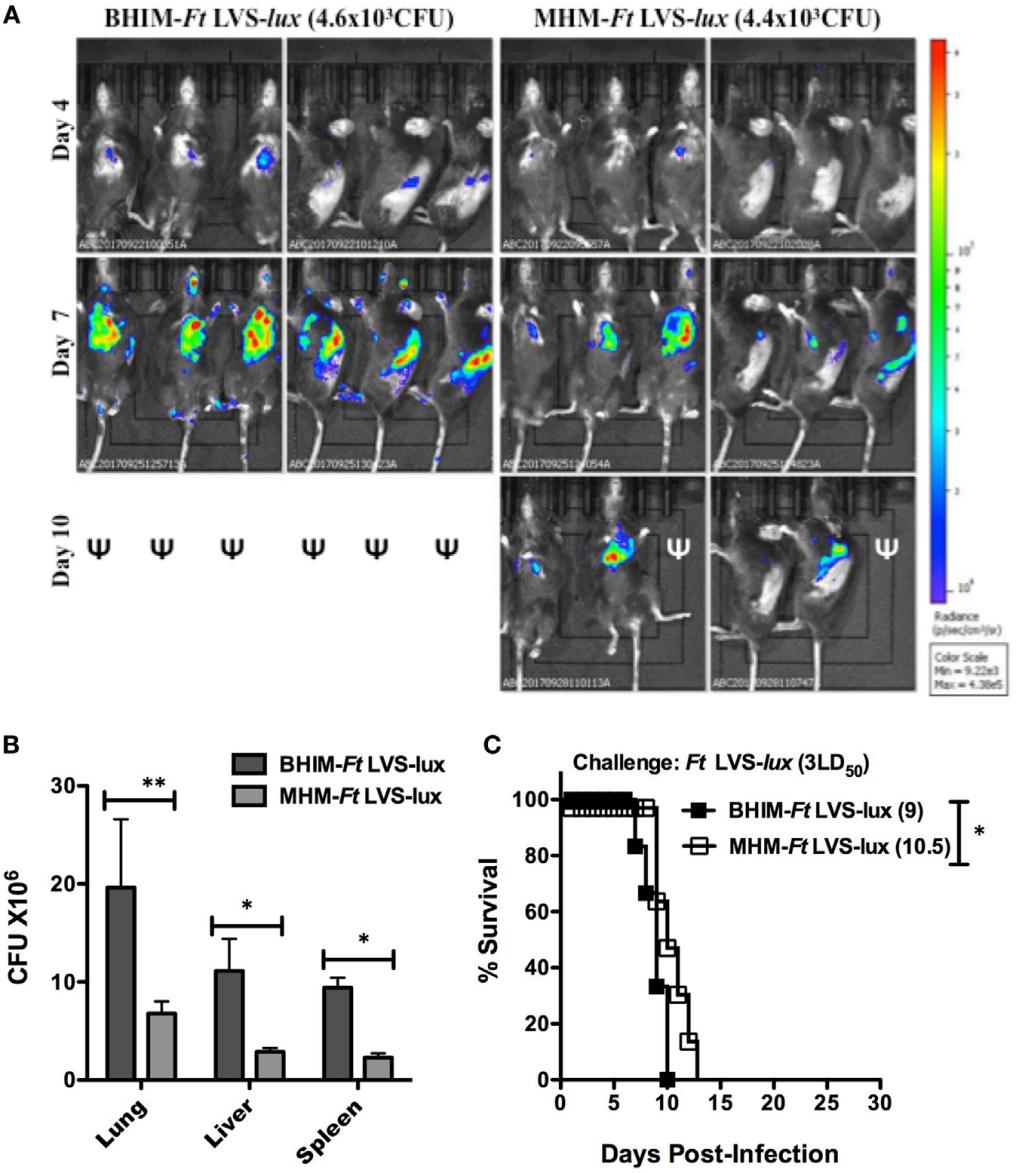
Brain-heart infusion medium (BHIM)-grown *Francisella tularensis* (*Ft*) replicate better *in vivo* than Mueller-Hinton medium (MHM)-grown *Ft*. Naïve C57BL/6 male and female mice (3/group) were anesthetized and then infected i.n. with either *Ft* LVS-*lux* grown in BHIM or MHM. Dissemination of *Ft* was monitored using *in vivo* imaging system Lumina on days 3 through 10 post-infection. Mice were imaged in ventral and side alignments. The image scaling was normalized by converting total counts to photons per second. The scale from blue to red represents low to high radiance efficiency expressed as p/sec/cm^2^/sr. ψ: indicates that mice were dead by the indicated time point post-infection **(A)**. Two groups of male and female mice were infected i.n. with 3LD_50_ BHIM or BHIM-*Ft* LVS-*lux* followed by analysis of bacterial burdens in lungs, liver, and spleen 7 days post-challenge **(B)**. Alternatively, mice were subsequently monitored for survival **(C)**. Median survival times (days) are specified next to the survival curves. *p < 0.05.

To determine the influence of growth medium on the course of tularemia infection, we performed a kinetic IVIS imaging analysis. Groups of three mice were infected i.n. with approximately 4.5 × 10^3^ CFU of BHIM or MHM-*Ft* LVS-*lux*. At 3 days post-infection (dpi), the mice displayed no clinical signs of infection and no bioluminescent signal was detected. At 4 dpi, mice infected with BHIM-*Ft* LVS-*lux* showed mild clinical signs and bioluminescent signals were detected in the lungs and spleens. At this time very little luminescent signal was observed emanating from the lungs of mice infected with MHM-*Ft* LVS-*lux*. On day 7 dpi, the imaging studies displayed a clear difference in the bioluminescent signal of BHIM-*Ft* LVS-*lux* versus MHM-*Ft* LVS-*lux*-infected mice (Figure 1A) with a significant difference in the amount of bioluminescence emitted from the ROI 6.41 × 10^6^ versus 1.35 × 10^6^ p/s/cm^2^/sr. Furthermore, the BHIM-*Ft* LVS-*lux* infection showed severe systemic dissemination from the lungs to the liver and spleen in each of these mice and most of the mice succumb to infection by day 10. In contrast, the MHM-*Ft* LVS-*lux*-infected mice exhibited mild dissemination and mortality. The increased bioluminescence in mice infected with BHIM-*Ft* LVS-*lux* was also evident in terms of higher bacterial burden and mortality (Figures 1B,C). A statistically significant difference in time to death was also observed between mice infected with BHIM-*Ft* LVS-*lux* (MTD of 9 days) and those infected with the MHM-*Ft* LVS-*lux* (MTD of 10.5 days). Collectively, BHIM-*Ft* displayed enhanced intracellular replication, dissemination, and virulence, as compared to its MHM-*Ft* counterpart.

### BHIM-*Ft* Generates Superior Protection Against *Ft* SchuS4 Challenge

**Figure 2:**
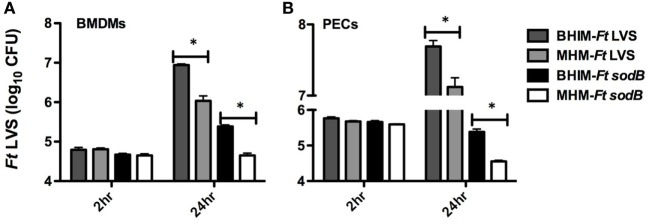
Differential intracellular growth kinetics of brain-heart infusion medium (BHIM)-*Francisella tularensis* (*Ft*) versus Mueller-Hinton Medium (MHM)-*Ft*. BMDMs **(A)** or resident peritoneal macrophages **(B)** from naive C57BL/6 male or female mice were incubated at an multiplicity of infection of 50 for 2 h with *Ft* (LVS or *sodB*) grown in either BHIM or MHM. After *Ft* infection, intracellular replication was quantified by plating and counting CFU at 2 and 24 h post-infection. Data show mean values and SEM from three independent experiments. *p < 0.05.

**Figure 3:**
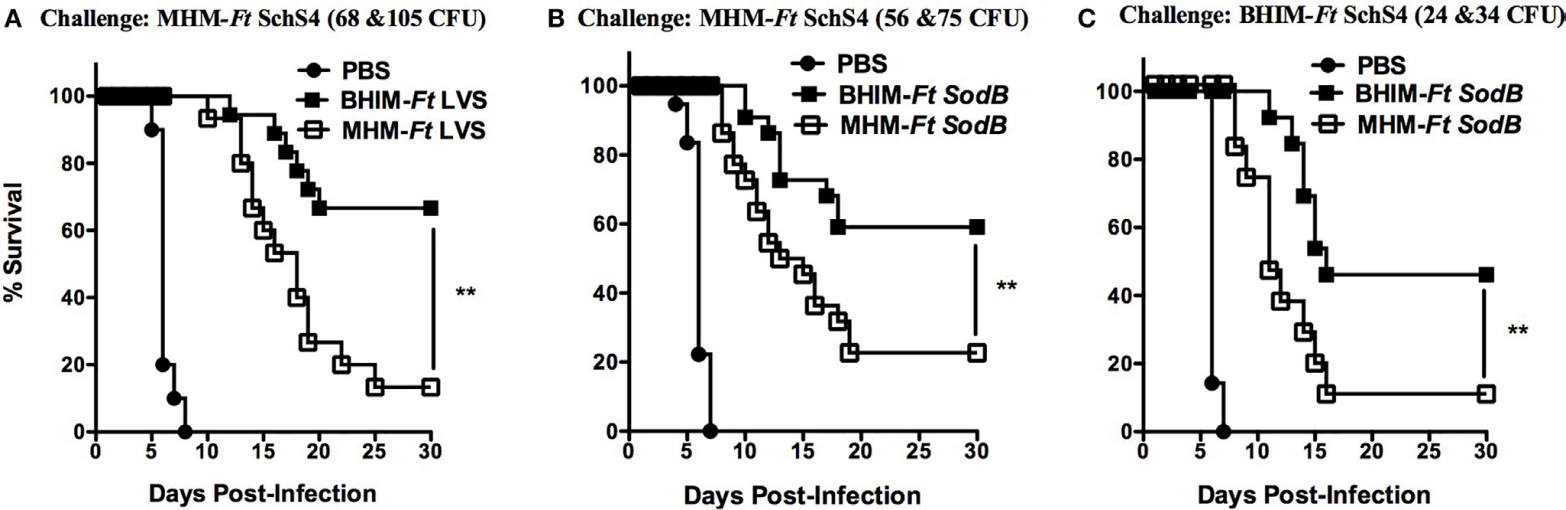
Vaccination with live brain-heart infusion medium (BHIM)-*Francisella tularensis* (*Ft*) provides better protection against *Ft* SchuS4 challenge C57BL/6 male and female mice were immunized i.d. with either 50 µl of vehicle (PBS) or 50 µl of 1 × 10^3^ CFU of BHIM versus Mueller-Hinton medium (MHM)-*Ft* LVS **(A)** or BHIM versus MHM-*Ft sodB* **(B,C)** on day 0 and boosted i.n. on day 21 with either 20 µl of vehicle (PBS) or 20 µl of 1 × 10^3^ CFU of BHIM versus MHM-*Ft* LVS or BHIM versus MHM-*Ft sodB*. Mice were then challenged i.n. on day 42 using MHM **(A,B)** or BHIM **(C)** -*Ft* SchuS4 and subsequently monitored for 30 days for survival. Data shown are representative of two independent experiments. **p < 0.01.

Having previously observed differences in protection following MHM-i*Ft* versus BHIM-i*Ft* vaccination and subsequent *Ft* LVS challenge (5), we sought to further evaluate whether cultivation of *Ft* in BHIM versus MHM has a similar impact on the protective efficacy of live *Ft* vaccine against challenge with the highly virulent category A select agent *Ft* SchuS4. To achieve a level of protection, which would allow us to make such a determination, we employed two strategies: first, mice were immunized with an i.d. prime followed by an i.n boost, which we have observed tends to generate a more protective response when using *Ft* LVS as the live vaccine. We also utilized *Ft* SodB, which is an attenuated form of *Ft* LVS and has previously been shown to provide clearly measurable protection against *Ft* SchuS4 infection in C57BL/6 mice (22). We first determined if the *ex vivo* growth kinetics of *sodB Ft* was impacted by growth conditions/medium similar to that of *Ft* LVS (3). To accomplish this, we performed an *ex vivo* intracellular infection/growth assay. Following macrophage exposure to *Ft*, the number of bacteria at 2 h was similar for BHIM or MHM-*Ft* (LVS or *SodB*) in all experiments performed, whether using BMDMs or RPMs (Figure 2). However, by 24 h post-infection there was a significant increase in the intracellular replication of the BHIM-*Ft* LVS in BMDMs (8.7 × 10^6^ CFU) compared to that of MHM-*Ft* LVS (1.7 × 10^6^ CFU) (Figure 2A). Importantly, *Ft sodB* showed a similar trend in the case of BMDMs and BHIM-*Ft* versus MHM-*Ft sodB* (2.4 × 10^5^ versus 4.4 × 10^4^ CFU). Results were analogous with RPMs (Figure 2B). However, despite using a similar MOI, there was substantial reduction in *Ft sodB* bacterial density compared to that of *Ft* LVS, as indicated by a 2–3-log reduction in *Ft* recovery (Figures 2A,B) compared to *Ft* LVS This impact on the growth of *Ft sodB* has also been observed during *in vivo* infection, providing additional supporting evidence for *Ft sodB* attenuation *in vivo* (23). In regard to challenge studies, mice were immunized with *Ft* LVS or *Ft SodB* grown in BHIM or MHM. Following immunization with PBS or 1 × 10^3^ CFU of wild-type *Ft* LVS generated in BHIM or MHM, and a lethal challenge with 68 or 105 CFU *Ft* SchuS4 (MHM), we observed that BHIM-*Ft*-immunized mice were better protected than MHM-*Ft*-immunized mice (67 versus 13%) (Figure 3A). Having observed a significant difference in protection with *Ft* LVS live vaccine, we then immunized mice with PBS or 1 × 10^3^ CFU of live attenuated vaccine (*Ft sodB*) grown in BHIM or MHM. Similar to that of *Ft* LVS vaccination, we observed superior (60%) protection against 56 and 75 CFU *Ft* SchuS4 challenge in the case of BHIM-*Ft sodB*-immunized mice, as opposed to poorer (23%) protection observed for MHM-*Ft sodB*-vaccinated mice (Figure 3B). To assess more rigorously the impact of differential cultivation on *Ft* vaccination and to evaluate any potential bias of growth medium used to culture the challenge organism on the outcome of survival, we similarly immunized mice with BHIM-*Ft sodB* or MHM-*Ft sodB*, then challenged with BHIM-*Ft* SchuS4. Again, we observed that BHIM-*Ft sodB* vaccination offered better (46%) protection compared to MHM-*Ft sodB*-vaccinated mice (9%) (Figure 3C). As expected based on previously published studies, survival rates among vaccinated mice challenged with BHIM-SchuS4 were lower than those of vaccinated mice challenged with MHM SchuS4 (4). Further, vaccination with either BHIM or MHIM-derived *Ft sodB* displayed similar trend of survival regardless of whether male or female mice are used (i.e., in both cases, whether looking at male or female, BHIM grown bacteria make better immunogens).

### Enhanced Protection Against *Ft* SchuS4 in BHIM-*Ft sodB*-Vaccinated Mice Is Associated With Reduced Bacterial Burden and Tissue Damage

**Figure 4:**
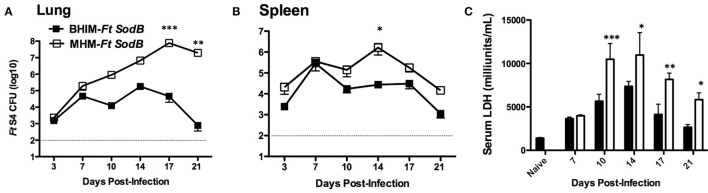
Enhanced protection against *Francisella tularensis* (*Ft*) SchuS4 challenge in brain-heart infusion medium (BHIM)-*Ft sodB-*vaccinated mice is associated with reduced bacterial burden and tissue damage. C57BL/6 female mice were immunized i.d. with either 50 µl of 1 × 10^3^ CFU of BHIM or Mueller-Hinton medium (MHM)-*Ft sodB* on day 0 and boosted i.n. on day 21 with 20 µl of 1 × 10^3^ CFU of BHIM or MHM-*Ft sodB*. Mice were then challenged i.n. on day 42 using MHM-*Ft* SchuS4. Bacterial burdens from lung **(A)** and spleen **(B)** were determined on days 3, 7, 10, 14, 17, and 21 post-challenge. The values represent the average bacterial count of three mice sacrificed at each time point ± SD and are from a single experiment. Similar results were obtained in two independent experiments. Serum lactate dehydrogenase concentrations **(C)** were quantified on indicated days post-challenge. Each bar represents mean ± SE (error bar) of two independent experiments with a total of six mice per group. *P < 0.05, **P < 0.01, and ***p < 0.001.

Further evidence of BHIM-*Ft sodB*-enhanced protection was provided when examining bacterial burden following immunization with BHIM-*Ft sodB* versus MHM-*Ft sodB* and subsequent challenge with *Ft* SchuS4. On days 3 and 7 post-challenge, there was no difference in the bacterial burden in the lungs of BHIM-*Ft sodB* and MHM-*Ft sodB*-vaccinated mice. However, by day 10 a ~60-fold reduction in bacterial burden was detected in BHIM-*Ft sodB* compared to MHM-*Ft sodB*-immunized mice by day 10 (Figure 4A). Importantly, it was not until day 10 post-challenge that MHM-*Ft sodB*-vaccinated mice began to die (Figures 3B,C). Furthermore, the bacterial numbers recovered from the lungs at later time points were substantially lower in BHIM-*Ft sodB*-vaccinated mice, as compared to that of the same tissues of MHM-*Ft sodB*-vaccinated mice. Similarly, BHIM-*Ft sodB*-immunized mice displayed a more controlled bacterial burden with lower bacterial numbers in the spleen compared to their MHM-*Ft sodB* counterparts (Figure 4B).

To provide further support for the above-observed protection differences in response to *Ft* SchuS4 challenge following vaccination, tissue inflammation was also assessed in BHIM-*Ft sodB* or MHM-*Ft sodB*-vaccinated mice following *Ft* SchuS4 infection by measuring the serum concentration of LDH. Serum LDH levels are commonly used as a marker of tissue damage (24). Similar to the bacterial burden data, no significant differences in the serum LDH concentration between the BHIM-*Ft sodB* and MHM-*Ft sodB* groups were observed in the first week following challenge. However, beginning 10 days post-challenge MHM-*Ft sodB*-vaccinated mice displayed increased levels of LDH in serum, as compared to that of BHIM-*Ft sodB-*immunized mice, thus indicating more severe inflammation and tissue destruction (Figure 4C).

### BHIM-*Ft sodB*-Vaccinated Mice Exhibit Reduced *Ft* SchuS4-Induced Inflammatory Cytokine Production

**Figure 5:**
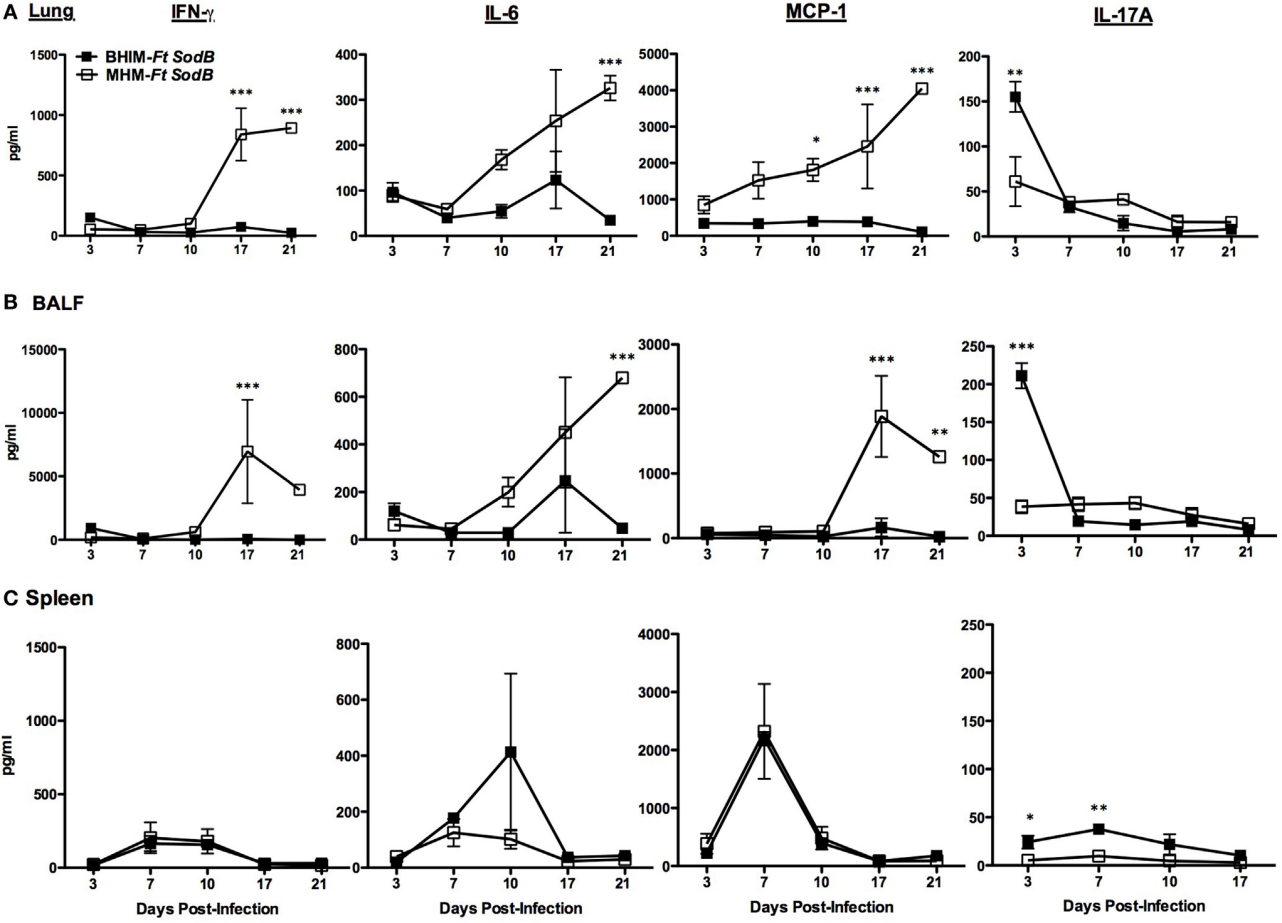
Brain-heart infusion medium (BHIM)- *Francisella tularensis* (*Ft*) *sodB*-vaccinated mice exhibit reduced *Ft* SchuS4-induced inflammation. C57BL/6 female mice were immunized with BHIM or Mueller-Hinton medium (MHM)-*Ft sodB* and challenged as described in Figure 4. The levels of pro-inflammatory cytokines from lung tissue homogenates **(A)**, BAL fluid **(B)**, and spleen homogenates **(C)** were determined on indicated days post-challenge. The values represent the average count of three mice sacrificed at each time point ± SD and are from a single experiment. Similar results were obtained in two independent experiments. *p < 0.05, **p < 0.01, and ***p < 0.001.

Our earlier vaccination studies confirmed that protected mice display higher levels of pro-inflammatory cytokines early on following *Ft* infection, as compared to unprotected mice (9, 21, 22). Conversely, in the present study, we observed that mice from both BHIM-*Ft sodB* and MHM-*Ft sodB*-immunized groups exhibited low levels of IFN-γ, IL-6, and MCP-1 (Figures 5A,B) in the lungs and BALF during the early stage of disease (up to day 10 post-infection). However, during later time points (after day 10 post-challenge) these pro-inflammatory cytokines were significantly elevated in MHM-*Ft sodB*-vaccinated mice, at which time majority of these mice succumb to infection (Figures 3B,C). This indicates that, controlled expression of pro-inflammatory cytokine levels is important for protection against systemic tissue damage and subsequent mortality inflicted by *Ft* infection. The levels of these pro-inflammatory cytokines in the spleens were comparable whether mice were immunized with BHIM-*Ft sodB* or MHM-*Ft sodB* (Figure 5C). Interestingly, we also observed that mice vaccinated with BHIM-*Ft sodB*, which are better protected, also exhibited higher levels of IL-17A at early time points [at day 3 (lungs and BALF) and day 7 (spleen) post-challenge], as compared to MHM-*Ft sodB*-immunized mice. Of note, the Th17 response has been found to correlate with protection against mucosal infections including tularemia (25). Collectively, survival, bacterial burden, inflammation, and inflammatory cytokine parameters all suggest that immunization with BHIM-*Ft sodB* is superior to that of MHM-*Ft sodB*.

### Immunization With BHIM-*Ft sodB* Induces Elevated Ab Responses

**Figure 6:**
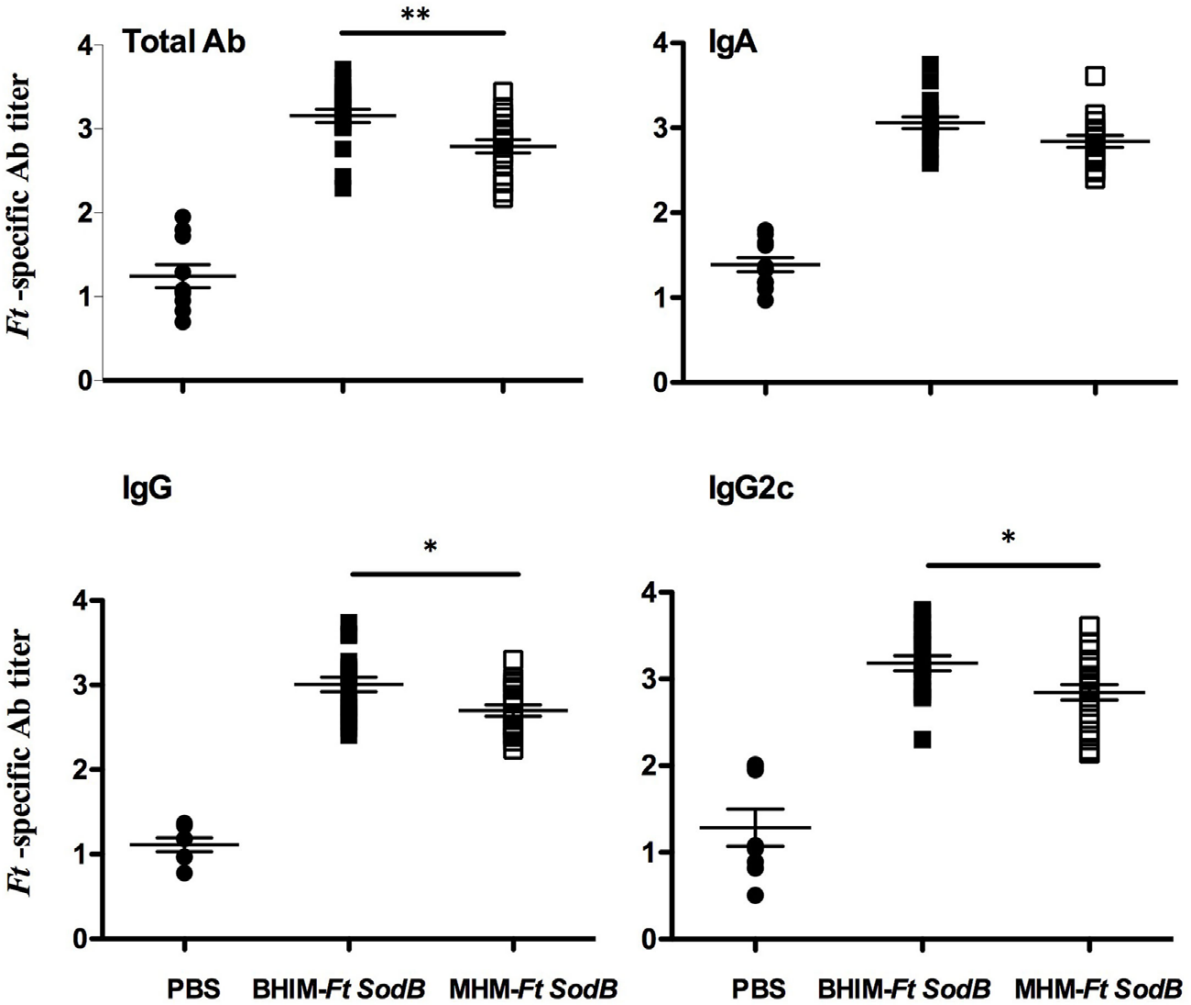
Immunization with brain-heart infusion medium (BHIM)-*Francisella tularensis* (*Ft*) *sodB* results in elevated Ab responses. C57BL/6 female mice were immunized with BHIM or MHM-*Ft sodB* as described in Figure 4. Sera obtained 3 weeks post-immunization were analyzed by enzyme linked immunosorbent assay (ELISA) for *Ft*-specific total Ab, IgA, IgG, and IgG2c Ab titers. Values represent mean ± SE of two combined experiments (*n* = 20 mice per group). Error bars represent SEM. *p < 0.05 and **p < 0.01.

Next, we wanted to determine whether *sodB Ft* grown in BHIM versus MHM has a differential impact on the humoral immune response, which, in some cases, can contribute to protection against *Ft* (21, 26). We quantified the *Ft*-specific Ab, including IgA, IgG, and IgG2c, in the serum of immunized mice and observed that BHIM-*Ft sodB* vaccination generated elevated levels of *Ft*-specific IgG and IgG2c, as compared to that of MHM-*Ft sodB*. However, *Ft*-specific IgA levels were similar for both groups (Figure 6).

### Immunization With BHIM-*Ft sodB* Induces Elevated T Effector Cell Ratios

**Figure 7:**
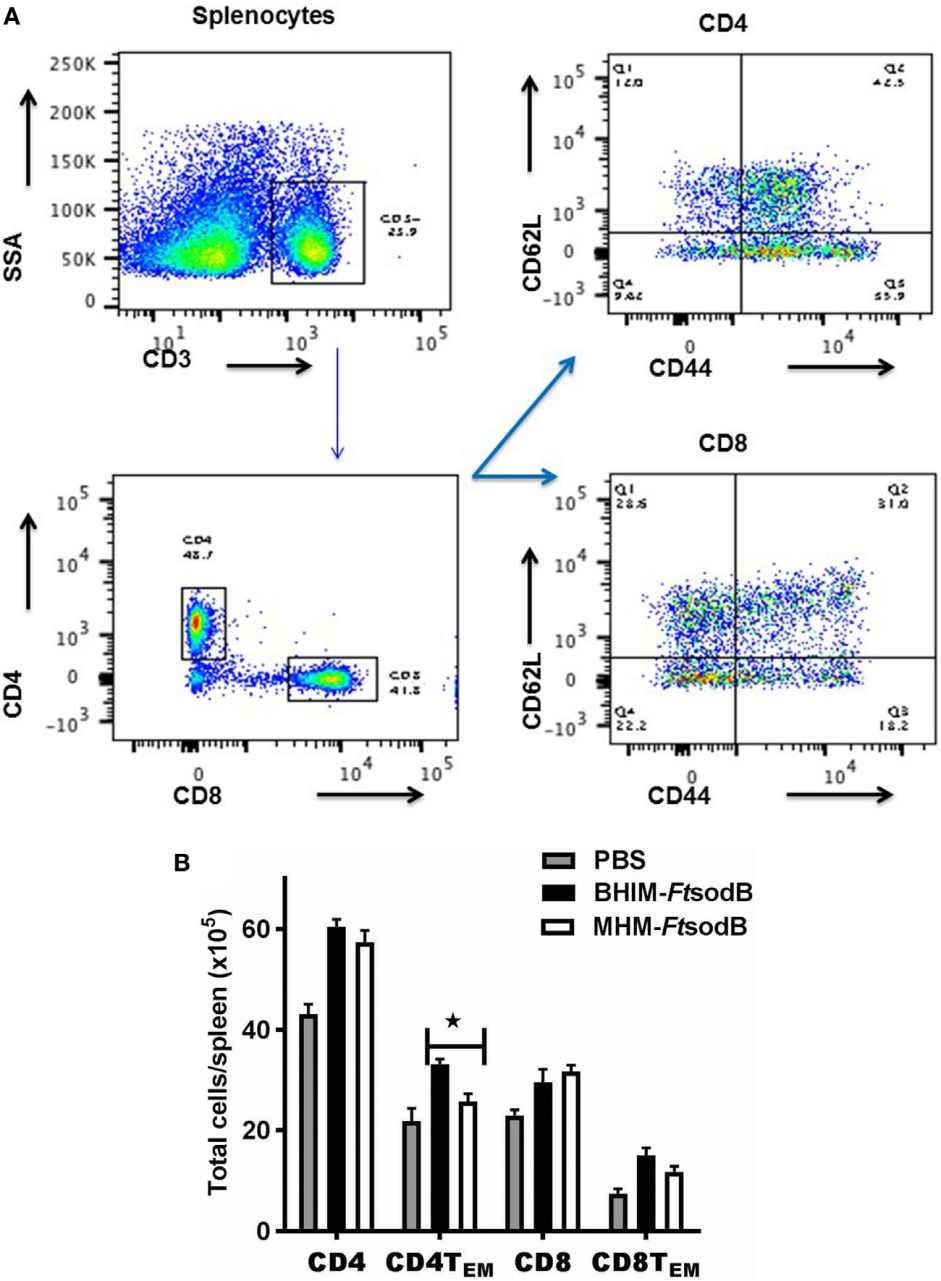
Immunization with brain-heart infusion medium (BHIM)-*Francisella tularensis* (*Ft*) *sodB* results in higher ratios of T effector cells. C57BL/6 female mice were immunized with BHIM or Mueller-Hinton medium (MHM)-*Ft sodB* as described in Figure 4. Single-cell suspensions of splenocytes from PBS or *sodB Ft* immunized mice (*n* = 3) were generated. Cells were counted and then stained for surface expression of CD4, CD8, CD44, and CD62L and analyzed by FACS. Representative scatterplots demonstrating gating strategy **(A)**, absolute cell counts of effector memory (CD44^hi^ CD62L^lo^) CD4^+^ and (CD44^hi^ CD62L^lo^) CD8^+^ T_EM_ cells percentage were determined **(B)**. *P < 0.05 compared to MHM-*Ft sodB*-immune mice. Data are representative of two independent experiments.

In addition to Ab response, cellular immunity is critical for intracellular pathogen containment and *Ft* control (27, 28). Specifically, CD4^+^and CD8^+^ memory T cells are considered the primary mediators of long-lived protection against *Ft* infection (29). Given the ability of BHIM-*Ft sodB* to protect mice against a lethal mucosal *Ft* SchuS4 infection, we postulated that mice vaccinated with BHIM-*Ft sodB* would exhibit higher numbers of effector memory T (T_EM_) cells. T_EM_ cells are characterized by reduced expression of the lymphoid tissue homing-associated marker (CD62L^low^) and augmented expression of source of inflammation homing-associated marker (CD44^hi^) (Figure 7A), as well as a tendency toward increased effector functions, including killing of infected cells and cytokine production (30). Mice immunized with either BHIM-*Ft sodB* or MHM-*Ft sodB* both exhibited higher CD4 and CD8 cells in the spleen, as compared to unvaccinated mice. However, there were significantly higher percentages of CD4 T_EM_ but comparable CD8 T_EM_ cell phenotypes from the BHIM-*Ft sodB*-immunized mice versus that of MHM-*Ft sodB*-immunized mice (Figure 7B). Altogether, these data demonstrate that there are qualitative and quantitative differences between BHIM-*Ft sodB* and MHM-*Ft sodB*-immunized mice with regards to CD4 cell phenotypes.

### Immunization With BHIM-*Ft sodB* Generates a Superior Memory/Recall Response

**Figure 8:**
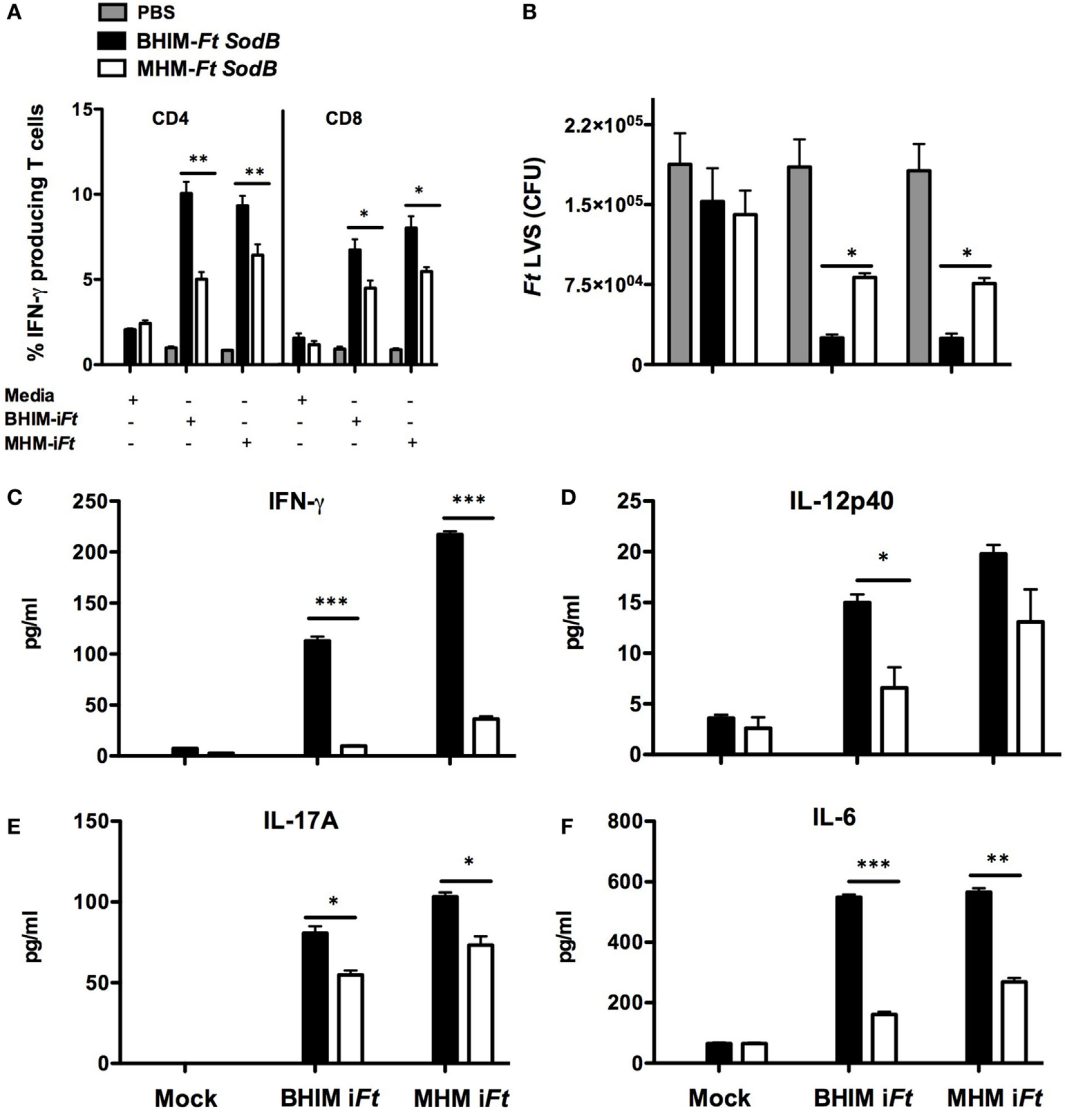
sodB-brain-heart infusion medium (BHIM)-*Francisella tularensis* (*Ft*)-immunized mice exhibit superior memory recall response. C57BL/6 female mice were immunized with BHIM or Mueller-Hinton medium (MHM)-*Ft sodB* mutant as described in Figure 4. Single-cell suspensions of splenocytes from PBS or *sodB*-mutant immune mice (*n* = 3) were stimulated overnight with BHIM or MHM-i*Ft*. The percentage of IFN-γ-producing CD4^+^ and CD8^+^ T cells was visualized by FACS **(A)**. *Ft* LVS was added to BMDM cultures containing PBS, BHIM-*Ft sodB*, or MHM-*Ft sodB*-immune splenocytes and stimulated overnight with BHIM or MHM-i*Ft*. The BMDMs were then lysed 72 h post-infection, diluted 10-fold, and plated on MH-chocolate agar plates to determine intracellular bacterial replication **(B)**. The accumulated levels of IFN-γ **(C)**, IL-12p40 **(D)**, IL-17A **(E)**, and IL-6 **(F)** in these cultures were quantified. The data presented are representative of two independent experiments. *p < 0.05, **p < 0.01, and ***p < 0.001.

To compare immune memory generated by BHIM-*Ft sodB* versus MHM-*Ft sodB*, we measured the recall response of T cells *ex vivo* by quantifying intracellular cytokine production by splenocytes from vaccinated and unvaccinated mice. Specifically, splenocytes from vaccinated mice were subjected to *in vitro* re-stimulation with i*Ft* grown in BHIM or MHM. Immune splenocytes from BHIM-*Ft sodB*-vaccinated mice re-stimulated with either BHIM or MHM-grown i*Ft* produced a significantly higher percentage of T-cells containing intracellular IFN-γ than re-stimulated, immune splenocytes from MHM-*Ft sodB*-vaccinated mice [CD4^+^ (9–10 versus 5–6.4%, respectively) and CD8^+^ (6.7–8 versus 4.5–5.5%, respectively)] (Figure 8A). T cell intracellular IFN-γ has been shown to correlate with improved vaccine efficacy (31–33). Similarly, we evaluated the capacity of splenocytes from BHIM-*Ft sodB* or MHM-*Ft sodB*-immunized mice to constrain the intracellular growth of *Ft* LVS under *ex vivo* conditions *via* a splenocyte-BMDM co-culture assay. The co-culturing of splenocytes from mice immunized with either BHIM-*Ft sodB* or MHM-*Ft sodB* immunogen resulted in increased growth inhibition, as compared to that of naive splenocytes. Concurrent with intracellular *Ft* growth inhibition, an enhanced intracellular killing of *Ft* LVS (2.5 × 10^4^ versus 8 × 10^4^ CFU/ml) was also observed in BMDMs co-cultured with BHIM-*Ft sodB*-immune splenocytes restimulated with either of BHIM- or MHM-grown i*Ft* (Figure 8B), suggesting that the enhanced efficacy of live BHI-*Ft* immunization is manifest at the vaccination stage. The prominent growth inhibition by BHIM-*Ft sodB*-immune splenocytes was further accompanied by the secretion of significantly higher levels of IFN-γ, IL-12, IL-17A, and IL-6 in cell culture supernatant, as compared to their MHM-*Ft sodB* counterparts (Figures 8C–F). Together, these results indicate that immune splenocytes obtained from BHIM-*Ft sodB*-immunized mice exhibit a more potent recall response *ex vivo* to *Ft* Ag than do immune splenocytes obtained from MHM-*Ft sodB*-immunized mice.

## Discussion

Given the large number of studies that have accomplished only moderate protection against a highly virulent *Ft* pulmonary challenge, it is clear that a number of important factors, including bacterial growth medium, must be further considered in *Ft* vaccine development. Specifically, environmental conditions, such as temperature, metal ions present, pH, and other media ingredients used for *in vitro* growth of bacteria have been shown to significantly influence antigen expression by bacterial pathogens (3–5, 11). Several studies have also further demonstrated a significant impact of growth conditions on immune function, such as bacterial recognition by Abs (13–15).

In the case of *Ft*, it has also been demonstrated that growth medium influences directly or indirectly the virulence of *Ft*, including its intracellular replication and internalization into lung and liver epithelial cells (34, 35). Accordingly, we found that both BHIM-*Ft* LVS and BHIM-*Ft sodB* exhibited an initial growth advantage *ex vivo* that manifests as more rapid intracellular growth compared to MHM-*Ft* (Figures 2A,B). BHIM-*Ft* also exhibits more rapid bacterial expansion *in vivo* with systemic dissemination leading to a slightly shorter mean survival time of naive animals infected with BHIM-*Ft* (Figures 1A–C) (3, 4, 11). Such differences also appear to be magnified in the context of pre-existing specific immunity, further emphasizing that the growth status of *Ft* is an important factor to be considered in vaccine immunogenicity and development.

Consistent with this, our vaccine studies also revealed that the two growth media (BHIM and MHM) employed for propagation of live *Ft* vaccines significantly altered the vaccine-induced immune response and protection against virulent *Ft* challenge. Specifically, BHIM-*Ft* (LVS or *sodB*)-immunized mice are significantly better protected against lethal SchuS4 challenge (Figures 3A–C). Interestingly, wild-type *Ft* LVS vaccination showed slightly better protection compared to *Ft* sodB mutant, which contradicts previous observations by Bakshi et al. (22). The possible reasons are (1) since we observed that current *Ft* LVS is more virulent (LD_50_:2,000 CFU) as compared to the parental stock (LD_50_:5,000 CFU) or that used by Bakshi et al. we reasoned that current *Ft* LVS could provoke superior protective immunity against *Ft* SchuS4. (2) Further, the modified immunization protocol, in the current study (i.d. prime followed by i.n boost), showed enhanced protection compared to that of i.n. prime followed by i.n boost used by Bakshi et al.

Consistent with the greater protective activity, the BHIM-*Ft sodB*-immune mice displayed reduced *Ft* burden accompanied by milder tissue inflammation. In MHM-*Ft sodB*-immunized mice a more robust expansion of *Ft* Schu4 and increased tissue inflammation in the lungs (Figures 4A–C) was evident. Most likely due to the latter, levels of pro-inflammatory cytokines IFN-γ, IL-6, and MCP-1 were also exacerbated in MHM-*Ft sodB*-immune mice late in infection (Figures 5A,B). In fact, it is well established that mice, which fail to protect against *Ft* challenge exhibit dysregulated production of pro-inflammatory cytokines as we observed and is described above (9, 21, 23). Interestingly, the aforementioned cytokines, which are indicators of systemic inflammation, illness, and sepsis (36), were substantially better controlled in BHIM-*Ft sodB*-immunized mice *Ft* SchuS4 challenge. In addition, levels of IL-17A were significantly higher in BHIM-*Ft sodB*-immunized mice at early time points following *Ft* SchuS4 challenge. Importantly, although IL-17A has been shown to be dispensable for host immunity to type A *Ft* infection (25), it also has been implicated as playing a role in protection against other mucosal infections including that of *Ft* LVS (37, 38).

We had hypothesized that the efficacy of *Ft* live vaccine would be impacted by the growth medium in which *Ft* is propagated. We further believed that *Ft* propagated in BHIM, which antigenically mimic bacteria propagated in macrophages, would be a better live vaccine immunogen than MHM-*Ft*. Accordingly, this was the case in that BHIM-*Ft sodB*-immune mice exhibited higher levels of *Ft*-specific IgG and IgG2c Abs. While early observations suggested that cellular immunity plays a more critical role in protection than Ab-mediated responses (26) & (Sunagar R and Gosselin, E. J. unpublished data), it has been reported that Ab and cell-mediated immune responses can also act synergistically in providing protection against virulent *Ft* infection (26, 27). Nevertheless, protection against *Ft*, as well as many other intracellular pathogens, is critically dependent on cell-mediated immunity (28). In this regard, a hallmark of successful vaccination is the generation of an effector memory response, which involves T_EM_ cells migrating to inflamed peripheral tissues and rapidly displaying effector function (30, 39). Specifically, superior protection of *Ft* LVS-vaccinated C57BL/6 mice *in vivo* has been correlated with an increased ratio of pulmonary and splenic T_EM_ cells (32). Consistent with these observations, we observed that spleens from the BHIM-*Ft sodB*-immune mice contained a considerably higher percentage of CD4 T_EM_ cells than those from MHM-*Ft sodB*-vaccinated mice. In addition, much emphasis has been placed on the role of the T cell cytokine IFN-γ as a correlate of protection in *Ft* infection (21, 40–42). Fittingly, our recall response studies demonstrate that the relative frequencies of CD4^+^ and CD8^+^ T cells expressing IFN-γ was considerably higher among BHIM-*Ft sodB* versus MHM-*Ft sodB*-immune splenocytes. Finally, our splenocyte-BMDM co-culture assay also revealed a role for enhanced bacterial killing (31–33). Splenocytes derived from the BHIM-*Ft sodB*-immune mice also produced more IFN-γ, IL-12p40, IL-17A, and IL-6 during BMDMs-splenocyte co-culture. This also supports prior studies suggesting that increased production of IFN-γ, IL-12p40, IL-17A, and IL-6 may serve as biomarkers for identifying more efficacious vaccine strategies against *Ft* (31, 33, 43).

It is also important to note that in our previous study, we observed differential protective efficacy of BHIM- and MHM-grown i*Ft* vaccine in which vaccination with MHM-i*Ft* provided better protection against mucosal infection with *Ft* LVS than BHIM-i*Ft*, which is in direct contrast to our results in this manuscript using live *Ft* LVS and *Ft sod*B vaccine (5). There may be a number of potential explanations for this dichotomy, which we are currently investigating. They include: differences in vaccine preparation, the vaccination regime, variations in the immune response to live versus inactivated vaccine, or differential immunological requirements for protection against *Ft* LVS and *Ft* SchuS4, taking into account that the prior studies with i*Ft* immunogen utilized *Ft* LVS challenges versus *Ft* SchuS4 challenges primarily used in these studies. Nevertheless, these studies strongly emphasize the critical importance of growth conditions when developing whole cell vaccines against *Ft* and very likely other bacterial pathogens as well.

## Ethics Statement

This study was conducted in agreement with the recommendations of the Institutional Animal Care and Use Committee (IACUC) of Albany Medical Center, Albany, NY, USA. The protocol was approved by the IACUC of Albany Medical Center, Albany, NY, USA.

## Author Contributions

RS, EG, and KH conceptualized and designed the study. RS, SK, and SR performed the experiments and acquired and analyzed the data. RS drafted the manuscript. EG and KH critically revised the manuscript. All the authors approved the publication of the manuscript and agreed to be accountable for all aspects of the work.

## Conflict of Interest Statement

The authors declare that the research was conducted in the absence of any commercial or financial relationships that could be interpreted as a potential conflict of interest.
